# Not All Probability Density Functions Are Tomograms

**DOI:** 10.3390/e26030176

**Published:** 2024-02-20

**Authors:** Liubov A. Markovich, Justus Urbanetz, Vladimir I. Man’ko

**Affiliations:** 1 Instituut-Lorentz, Universiteit Leiden, P.O. Box 9506, 2300 RA Leiden, The Netherlands; j.urbanetz@googlemail.com; 2 Institute for Information Transmission Problems, Bol. Karetny per. 19, Moscow 127051, Russia; 3 Russian Quantum Center, Skolkovo, Moscow 121205, Russia; mankovi@lebedev.ru; 4 Lebedev Physical Institute, Russian Academy of Sciences, Leninskii Prospect 53, Moscow 119991, Russia

**Keywords:** probability distribution function, symplectic tomogram, quantum state reconstruction, characteristic function

## Abstract

This paper delves into the significance of the tomographic probability density function (pdf) representation of quantum states, shedding light on the special classes of pdfs that can be tomograms. Instead of using wave functions or density operators on Hilbert spaces, tomograms, which are the true pdfs, are used to completely describe the states of quantum systems. Unlike quasi-pdfs, like the Wigner function, tomograms can be analysed using all the tools of classical probability theory for pdf estimation, which can allow a better quality of state reconstruction. This is particularly useful when dealing with non-Gaussian states where the pdfs are multi-mode. The knowledge of the family of distributions plays an important role in the application of both parametric and nonparametric density estimation methods. We show that not all pdfs can play the role of tomograms of quantum states and introduce the conditions that must be fulfilled by pdfs to be “quantum”.

## 1. Introduction

In the early 1900s, Schrödinger introduced the concept of a quantum system’s state [1], characterised by a complex wave function. The notion of a density matrix [2,3,4] or a density operator, acting in a Hilbert space, was established shortly after to characterise the generic states of quantum systems.

Nowadays, quantum state reconstruction is a critical task in quantum information science and quantum technology. We know that, in classical physics, it is always possible to assign a unique probability distribution function (pdf) to the outcomes of a physical measurement in continuous-variable systems. Based on the measurement results, one can estimate the pdf using different parametric and nonparametric methods [5] and, thus, fully describe the system. However, in quantum mechanics, the Heisenberg uncertainty principle states that certain pairs of non-commuting observables, such as position and momentum, cannot be simultaneously precisely determined. Therefore, it is challenging to reconstruct the quantum state from measurements, which leads to the need for a more sophisticated representation of the quantum state.

Quasi-probability distributions (qpdfs) are introduced to represent the probabilities of outcomes of non-commuting observables, such as position and momentum, in a way that respects the uncertainty principle. Qpdfs are not actual probabilities in the classical sense. These distributions are often complex-valued and can take negative values, which can make their interpretation challenging. The Wigner function is one of the most well-known qpdfs [6]. It provides a phase-space representation of a quantum state, allowing the simultaneous description of position and momentum. The Wigner function is real-valued and can take negative values. The Husimi Q-function is another qpdf [7,8] that is positive and can be thought of as a smoothed version of the Wigner function [9]. The Glauber–Sudarshan P-function [10,11] is used to represent states in the context of quantum optics and can be used for the description of coherent states. There exist other quasi-probabilities defined as different Fourier-like transforms, like Kirkwood–Rihaczek (KR) [12], Generalised KR [13], Margenau–Hill (MH) [14], Page [15], Choi–Williams (CW) [16], sinc [17], and Cohen [18]. A symplectic tomogram [19,20] is an alternative way to represent the quantum state. It is a real-valued continuous probability distribution in the phase space and is constructed through a convolution of the density operator with a kernel that depends on the phase space coordinates. The resulting function represents the probability of finding the quantum system in a particular position and momentum configuration. The symplectic tomogram is related to the qpdfs with invertible integral transforms [21]. In fact, the Wigner function can be derived from the symplectic tomogram through an inverse Radon transform [22].

Wigner functions can take negative values, which makes them incompatible with classical probability distributions. The function negativity leads to difficulties in interpreting the reconstructed state, especially when attempting to represent it as a physical probability distribution. Since the symplectic tomogram is a pdf, one can use all the techniques known in probability theory for pdf estimation. Furthermore, the measurable random variables are generated by a distribution which, as we see, is the tomogram. There is no point in reconstructing the Wigner function and then the state if the tomogram estimate can be immediately used for state estimation. The parametric pdf estimation methods strongly rely on the knowledge of the class of the pdf. For example, the histogram method is widely used in practise [23,24] because of its simplicity. It divides the data range into intervals, or bins, and counts the number of data points in each bin. The pdf is then approximated by normalising these counts. However, in some cases, a data sample does not resemble a common probability distribution or cannot be easily made to fit the distribution. This is often the case when the data have two peaks (bimodal distribution) or many peaks (multi-modal distribution). In these cases, nonparametric methods are used. These methods do not assume a specific parametric form for the pdf. Hence, knowledge of the pdf family is essential for an optimal selection of the pdf reconstruction method.

By definition, a state is called Gaussian if its Wigner function is a Gaussian-like function [25]. Since the Wigner function and the tomogram are connected by the Radon transform, we can say that the state is Gaussian if its tomogram is a Gaussian pdf. In [26], the quantum harmonic oscillator (HO) is studied, showing that its tomograms are from the exponential family of distributions [27]. In particular, the tomogram of the ground state is the Gaussian pdf. The inverted oscillator [28] state tomogram is also from the exponential family. The tomograms are known to be Gaussian pdfs for the squeezed vacuum states and the thermal states [29]. However, a superposition of Gaussian states is already a non-Gaussian mixture [30]. There has been a strong experimental focus on two specific types of non-Gaussian states: cat states [31] and Gottesman–Kitaev–Preskill (GKP) [32] states. In Ref. [33], the crystallised cat state tomograms are presented. As the superposition of cat states, their resultant states are non-Gaussian too since their tomogram is the sum of Gaussian functions, which, in general, is not a Gaussian pdf.

Thus, non-Gaussian states are frequently encountered in practice. However, can entirely different distributions describe a quantum state, for instance, from the exponential or power-law family? It is evident that not all pdfs are suitable for the role of a tomogram due to the constraints imposed on the quantum-state density operator, e.g., that it is nonnegative, Hermitian, and has a unit trace. Thus, the question we address in this paper is: What continuous distributions can describe quantum states? Knowing the answer, we can solve the inverse problem: given a pdf playing the role of the tomogram, find the new corresponding quantum state not known in the literature.

### 1.1. Contributions of This Paper

In this manuscript, we introduce the characteristic function of the tomogram pdf as a description of the quantum state. Given the Fourier transform of the pdf, the characteristic function always exists and is bounded. We show that the characteristic function of the pdf corresponding to the quantum state must fulfil the conditions following from the density operator properties. If the distribution does not satisfy these conditions, then the corresponding quantum state does not exist. Thus, it is possible to assess the ability of known pdfs to generate physical quantum states.

The trace of the density operator and the purity parameter are explicitly written in terms of the characteristic function. Moreover, we show that the trace of the product of two density operators can be written as an integral from their characteristic functions corresponding to their tomogram pdfs. Similarly the fidelity parameter is written. Thus, knowledge about the characteristic function can be used for purity testing in the case of two identical states or in the case of two different states for testing their overlap. Also, the elements of the density matrix are written in terms of the characteristic function, giving a novel way to parametrise the density matrix.

We investigate the exponential family of pdfs to find the conditions on its parametrisation to satisfy the constraints on the characteristic function. We check some known pdfs from the exponential family: exponential, gamma, and $\chi^{2}$ pdfs, showing that they never satisfy the latter conditions, thus not being suitable for quantum-state generation.

Finally, we study particular systems with continuous variables like oscillators, the superposition of cat states, and different Gaussian states. Their pdfs either belong to the exponential family of pdfs or are a mixture of Gaussians. The corresponding characteristic functions are derived, showing that they satisfy the conditions imposed on the characteristic function to generate a physical quantum state. For a pseudoharmonic oscillator [34] that is defined on the positive semiaxis, we deduce the tomogram for the first time, showing it is from the exponential family too.

### 1.2. Organisation of the Paper

The paper is organised as follows. In Section 2, we recall the notion of the symplectic tomogram, giving its connection to the density matrix of the quantum state. In Section 3, the characteristic function of the tomogram is introduced. The requirements that must be satisfied to characterise a quantum state are outlined. In Section 4, the general exponential family of pdfs is considered. We study the examples of continuous pdfs known in classical probability theory. The quantum harmonic and pseudoharmonic oscillators are studied in detail in Section 5. The superposition of cat states is analysed in Section 6.

## 2. Symplectic Tomogram

Let us consider a quantum state in an infinite-dimensional Hilbert space H associated with a positive Hermitian operator $\hat{\rho }$, called the density matrix. The kernel of the density matrix in coordinate representation is $\rho (x,x^{\prime })=\langle x|\rho |x^{\prime }\rangle$, where |x〉 is an eigenstate of the position operator $\hat{q}$. The density matrix operator is hermitian ($\rho^{★}\left(x,x^{\prime }\right)=\rho \left(x^{\prime },x\right)$). Its diagonal elements are nonnegative (ρ(x,x)≥0) and its trace is equal to one (∫ρ(x,x)dx=1). Unlike the wave function, which is only suitable for describing pure states, the density operator can equally represent both pure and mixed states.

According to the general scheme [19], in the case of continuous variables, the mapping of the density matrix to the family of pdfs, depending on two real parameters μ and ν, is given by the following relation:

(1) $$\begin{array}{c} W\left(X|\mu ,\nu \right)=\mathrm{Tr}\left(\hat{\rho }\delta \left(\hat{1}X-\mu \hat{q}-\nu \hat{p}\right)\right)=\langle X;\mu ,\nu |\hat{\rho }|X;\mu ,\nu \rangle . \end{array}$$

δ(·) is the Dirac delta function. Here, $\hat{q}$ and $\hat{p}$ are the position and momentum operators:

(2) $$\begin{array}{c} \hat{q}|X;\mu ,\nu \rangle =X|X;\mu ,\nu \rangle , \end{array}$$

and |X;μ,ν〉 is an eigenvector of the hermitian operator:

(3) $$\begin{array}{c} \hat{X}\left(\mu ,\nu \right)=\mu \hat{q}+\nu \hat{p},\;\hbar =1. \end{array}$$

The latter is a canonical transform of $\hat{q}$ and $\hat{p}$. Formally, this quantity is a coordinate, measured in a scaled and rotated reference frame in the phase space. Its pdf (1) is called the *symplectic tomogram*. As a pdf, the tomogram is nonnegative and normalised:

(4) $$\begin{array}{c} \int_{-\infty }^{\infty }W\left(X|\mu ,\nu \right)dX=1. \end{array}$$

For the symplectic tomogram, the inverse quantum Radon transform is the following:

(5) $$\begin{array}{c} \hat{\rho }=\frac{1}{2\pi }\int W\left(X|\mu ,\nu \right)\exp\left(i\left(X\hat{1}-\mu \hat{q}-\nu \hat{p}\right)\right)dXd\mu d\nu , \end{array}$$

defining the density matrix operator by the corresponding pdf. For the density operator $\hat{\rho }=|\psi \rangle \langle \psi |$ of the pure state |ψ〉, Relation (1) converts into

(6) $$\begin{array}{c} W\left(X|\mu ,\nu \right)=\frac{1}{2\pi |\nu |}|\int_{-\infty }^{\infty }\Psi \left(y\right)\exp\left(\frac{i\mu }{2\nu }y^{2}-\frac{iX}{\nu }y\right)dy{|}^{2}. \end{array}$$

The representation in (5) is connected with the Weyl and star-product quantisation [22]. It is known that the Weyl symbol of the density matrix is explicitly the Wigner function. It is shown in [20] that the symplectic tomogram is related to the quantum state expressed in terms of its Wigner function W(q,p) with an integral transform. The parameters μ and ν describe an ensemble of rotated and scaled reference frames, in which the observable *X* is measured. For μ=cosϕ and ν=sinϕ, the pdf W(X|μ,ν) is the distribution for homodyne output variable used in optical tomography [35,36]. The procedure of balanced homodyne photon detection is based on mixing of a measurable (weak) field and a strong coherent field with varying phase ϕ on the beam splitter. In this case, the measurable observable is $\hat{X}=\hat{q}\cos\phi +\hat{p}\sin\phi$. The angle ϕ could be interpreted as a rotation angle of the phase space. Relations (1)–(6) for a symplectic tomogram are transformed to equivalent relations for optical tomograms. Note that the symplectic tomogram is a function of two parameters (μ,ν) of the Sp(2,R) group parameterisation, and the optical tomogram is a function of the parameter ϕ.

Alternatively, the parameters μ and ν can be expressed in the form scosϕ, $s^{-1}\sin\phi$, where s>0 is a real squeezing parameter and ϕ is a rotation angle. Then, the variable *X* is identical to the position measured in the new reference frame in the phase space with axes sq and $s^{-1}p$, and, after the scaling, the axes are rotated by an angle ϕ. Thus, the tomogram implies the pdf of the random position *X* measured in the new (scaled and rotated) reference frame in the phase space [37].

The process of inverting the raw observed data in order to arrive at a form of the quantum state is both delicate and intriguing. Deterministic and nondeterministic inversion methods are the two primary categories of inversion techniques. In the deterministic methods, an experimentally determined pdf (tomogram) is used to determine the matrix elements of the density matrix by a direct mathematical inversion of (5). However, this strongly depends on how well we estimate the pdf based on the measurement of *X* for varying ϕ. The nondeterministic technique aims to directly estimate the quantum state, as opposed to using classical distributions as an intermediate object [38,39].

## 3. Characteristic Function

Since the tomogram is the pdf of the variable *X* depending on real parameters μ and ν, we can introduce its characteristic function. According to the definition, the characteristic function

(7) $$\begin{array}{c} \phi_{X}\left(t\right)\equiv \int_{-\infty }^{\infty }W\left(X\right)e^{itX}dX \end{array}$$

is the Fourier transform of the pdf. It is known that a random variable $X_{n}$ weakly converges to a random variable *X* if and only if, for any *t*, the characteristic function $\phi_{X_{n}}\left(t\right)$ converges to the characteristic function $\phi_{X}\left(t\right)$ [40]. Further, we omit *X* in the notation $\phi_{X}\left(t\right)$.

The characteristic function of any real-valued random variable completely defines its probability distribution. According to definition, it is non-vanishing in a region around zero (ϕ(0)=1) and it is bounded |ϕ(t)|≤1, ∀t. We can introduce the characteristic function ϕ(t;μ,ν) corresponding to the tomogram pdf (1):

(8) $$\begin{array}{c} \phi \left(t;\mu ,\nu \right)\equiv \int_{-\infty }^{\infty }W\left(X|\mu ,\nu \right)e^{itX}dX. \end{array}$$

Then, we can rewrite the density operator (5) as follows:

(9) $$\begin{array}{c} \hat{\rho }=\frac{1}{2\pi }\int \phi \left(1;\mu ,\nu \right)e^{-i(\mu \hat{q}+\nu \hat{p})}d\mu d\nu . \end{array}$$

and, taking the trace from both sides, we can conclude

(10) $$\begin{array}{c} \mathrm{Tr}\hat{\rho }=\int e^{i\frac{\mu \nu }{2}}\phi \left(1;\mu ,\nu \right)\delta \left(\mu \right)\delta \left(\nu \right)d\mu d\nu =\phi \left(1;0,0\right), \end{array}$$

where we use $Tr\left(e^{-i(\nu \hat{p}+\mu \hat{q})}\right)=2\pi e^{i\frac{\mu \nu }{2}}\delta \left(\mu \right)\delta \left(\nu \right)$. Thereby, the trace of the density matrix of a quantum state is the characteristic function with μ=0,ν=0. That gives us the condition on the characteristic function of the quantum state: ϕ(1;0,0)=1.

Using (5), we can write the product of two density matrices, ${\hat{\rho }}_{1}$ and ${\hat{\rho }}_{2}$, corresponding to two quantum states. Taking the trace, we obtain

(11) $$\begin{array}{ccc} \mathrm{Tr}\left({\hat{\rho }}_{1}{\hat{\rho }}_{2}\right) & = & \frac{1}{2\pi }\;\int \int \;\phi_{1}\left(1;\mu_{1},\nu_{1}\right)\phi_{2}\left(1;-\mu_{1},-\nu_{1}\right)d\mu_{1}d\nu_{1}, \end{array}$$

where $\phi_{1}\left(1;\mu_{1},\nu_{1}\right)$ and $\phi_{2}\left(1;\mu_{1},\nu_{1}\right)$ are the characteristic functions, corresponding to the tomograms of the states. Since the modulus of the characteristic function is bounded by one, we can be sure that the latter integral is also always bounded by one that fully coincides with the left-hand side trace upper bound. However, the integral can be negative since the characteristic function in general can take negative values. The left-hand-side trace from the product of two density matrices is always nonnegative, so the condition on the characteristic function of the quantum state holds:

(12) $$\begin{array}{c} 0\leq \frac{1}{2\pi }\overset{\infty }{\underset{-\infty }{\;\int \int \;}}\phi_{1}\left(1;\mu ,\nu \right)\phi_{2}\left(1;-\mu ,-\nu \right)d\mu d\nu \leq 1. \end{array}$$

When ${\hat{\rho }}_{1}={\hat{\rho }}_{2}$, holds, the left-hand side of (11) is the purity parameter:

(13) $$\begin{array}{ccc} \mathrm{Tr}\left({\hat{\rho }}^{2}\right) & = & \frac{1}{2\pi }\overset{\infty }{\underset{-\infty }{\;\int \int \;}}\phi \left(1;\mu ,\nu \right)\phi \left(1;-\mu ,-\nu \right)d\mu d\nu . \end{array}$$

Estimation of the overlap $\mathrm{Tr}\left({\hat{\rho }}_{1}{\hat{\rho }}_{2}\right)$ between two quantum states ${\hat{\rho }}_{1}$ and ${\hat{\rho }}_{2}$ is a frequently encountered task in quantum information science [41,42,43]. It provides a basis for deriving several important properties of quantum systems, including fidelity, purity, Hilbert–Schmidt distance, and Renyi entropy.

If one knows the estimates of the characteristic functions of the states, one can estimate the purity or the overlap between the density matrices. For ideally pure states ${\hat{\rho }}_{1}=|\psi_{1}\rangle \langle \psi_{1}|$ and ${\hat{\rho }}_{2}=|\psi_{2}\rangle \langle \psi_{2}|$, the trace distance is $\mathrm{Tr}\left({\hat{\rho }}_{1}{\hat{\rho }}_{2}\right)={\left|\langle \psi_{1}|\psi_{2}\rangle \right|}^{2}$, i.e., a direct measure of orthogonality of $|\psi_{1}\rangle$ and $|\psi_{2}\rangle$. The trace product $\mathrm{Tr}\left({\hat{\rho }}_{1}{\hat{\rho }}_{2}\right)$ is equal to zero if the states are orthogonal and equal to one if they are identical.

The result in (11) is easily extendable to the case of the product of *d* density matrices:

(14) $$\begin{array}{c} \mathrm{Tr}\left(\prod_{i=1}^{d}{\hat{\rho }}_{i}\right)\;=\;\frac{1}{{\left(2\pi \right)}^{d-1}}\overset{\infty }{\underset{-\infty }{\;\int \int \;}}\prod_{i=1}^{d-1}\phi_{i}\left(1;\mu_{i},\nu_{i}\right)\phi_{d}\left(1;-\sum_{i=1}^{d-1}\mu_{i},-\sum_{i=1}^{d-1}\nu_{i}\right)d\mu_{1}\ldots d\mu_{d-1}d\nu_{1}\ldots d\nu_{d-1}, \end{array}$$

where $\phi_{i}\left(1;\mu_{i},\nu_{i}\right)$ are the characteristic functions of the corresponding states ${\hat{\rho }}_{i}$, respectively. If all ${\hat{\rho }}_{i}$ are equal, then the latter result reduces to

(15) $$\begin{array}{c} \mathrm{Tr}\left({\hat{\rho }}^{d}\right)\;=\;\frac{1}{{\left(2\pi \right)}^{d-1}}\overset{\infty }{\underset{-\infty }{\;\int \int \;}}\prod_{i=1}^{d-1}\phi \left(1;\mu_{i},\nu_{i}\right)\phi \left(1;-\sum_{i=1}^{d-1}\mu_{i},-\sum_{i=1}^{d-1}\nu_{i}\right)d\mu_{1}\ldots d\mu_{d-1}d\nu_{1}\ldots d\nu_{d-1}, \end{array}$$

Transition amplitudes between different states play a significant role in quantum science. For example, let the system be in a pure state $|\psi_{1}\rangle$. Then, the transition amplitude to the pure state $|\psi_{2}\rangle$ is the scalar product of these two states. Consequently, the probability of transitioning from the initial pure state to another is the square modulus of the transition amplitude: $\omega_{\psi_{1}\to \psi_{2}}={\left|\langle \psi_{1}|\psi_{2}\rangle \right|}^{2}$. Thus, we can write

(16) $$\begin{array}{ccc} \omega_{\psi_{1}\to \psi_{2}} & = & \langle \psi_{1}|\psi_{2}\rangle {\left(\langle \psi_{1}|\psi_{2}\rangle \right)}^{★}=\langle \psi_{1}|\left(|\psi_{2}\rangle \langle \psi_{2}|\right)|\psi_{1}\rangle \\ & = & \mathrm{Tr}\left(|\psi_{2}\rangle \langle \psi_{2}||\psi_{1}\rangle \langle \psi_{1}|\right)=\mathrm{Tr}\left(\rho_{\psi_{2}}\rho_{\psi_{1}}^{†}\right). \end{array}$$

The latter transition probability is called fidelity for pure states. Using the characteristic function, this can be written as

(17) $$\begin{array}{c} \omega_{\psi_{1}\to \psi_{2}}=\frac{1}{2\pi }\int \phi_{2}\left(1;\mu ,\nu \right)\phi_{1}\left(-1;-\mu ,-\nu \right)d\mu d\nu . \end{array}$$

**Lemma** **1.** *(Hermiticity) The integral in (9) defines the hermitian operator if and only if the characteristic function satisfies the condition*

(18) $$\begin{array}{c} \phi (1;\mu ,\nu )=\phi (-1;\mu ,-\nu ),\;\forall \mu ,\nu . \end{array}$$

**Proof.**  Let $\hat{\rho }$ be a hermitian operator with a matrix ρ. Its matrix elements satisfy $\rho \left(y,y^{\prime }\right)=\rho^{★}\left(y^{\prime },y\right)$, $\forall y,y^{\prime }$, where ${\left(\cdot \right)}^{★}$ denotes the complex conjugation. One can write these matrix elements as follows (see the deduction in Appendix A):

(19) $$\begin{array}{ccc} \rho (y,y^{\prime })\;\; & = & \;\;\frac{1}{2\pi }\int \phi \left(1;\mu ,y-y^{\prime }\right)\exp\left(-i\frac{\mu (y+y^{\prime })}{2}\right)d\mu =\frac{1}{\sqrt{2\pi }}\hat{\phi }\left(1;\frac{y+y^{\prime }}{2},y-y^{\prime }\right),\\ \rho^{★}\left(y^{\prime },y\right)\;\;\;\; & = & \;\;\;\;\frac{1}{2\pi }\int \phi \left(-1;\mu ,y^{\prime }-y\right)\exp\left(-i\frac{\mu (y+y^{\prime })}{2}\right)d\mu =\frac{1}{\sqrt{2\pi }}\hat{\phi }\left(-1;\frac{y+y^{\prime }}{2},y^{\prime }-y\right), \end{array}$$

where $\hat{\phi }\left(1;\tilde{\mu },\tilde{\nu }\right)$ is a Fourier transform of ϕ(1;μ,ν). We can conclude that the characteristic function must satisfy

(20) $$\begin{array}{c} \phi \left(1;\mu ,y-y^{\prime }\right)=\phi \left(-1;\mu ,y^{\prime }-y\right),\;\forall y,y^{\prime }. \end{array}$$

or, in the form of the Fourier transform,

(21) $$\begin{array}{c} \hat{\phi }\left(1;\frac{y+y^{\prime }}{2},y-y^{\prime }\right)=\hat{\phi }\left(-1;\frac{y+y^{\prime }}{2},y^{\prime }-y\right),\;\forall y,y^{\prime }. \end{array}$$

Finally, we can conclude that (18) holds ∀μ,ν since $y,y^{\prime }$ are independent.

Now, let Condition (18) hold. Then, one can write

(22) $$\begin{array}{ccc} \rho (y,y^{\prime })\;\; & = & \;\;\frac{1}{2\pi }\int \phi \left(1;\mu ,y-y^{\prime }\right)\exp\left(-i\frac{\mu (y+y^{\prime })}{2}\right)d\mu \\ & = & \frac{1}{2\pi }\int \phi \left(-1;\mu ,y^{\prime }-y\right)\exp\left(-i\frac{\mu (y+y^{\prime })}{2}\right)d\mu \\ & = & \frac{1}{\sqrt{2\pi }}\hat{\phi }\left(-1;\frac{y+y^{\prime }}{2},y^{\prime }-y\right)=\rho^{★}\left(y^{\prime },y\right). \end{array}$$

□

**Lemma** **2.** *(Positivity) The integral in (9) defines the positive semi-definite operator $\hat{\rho }$ if and only if the characteristic function satisfies the condition*

(23) $$\begin{array}{c} \hat{\phi }\left(1;y,0\right)\geq 0,\;\forall y. \end{array}$$

**Proof.**  Let $\hat{\rho }$ be a positive semi-definite operator. Using the expression for $\rho (y,y^{\prime })$ from the previous proof, the diagonal elements of the density matrix can be written as

(24) $$\begin{array}{c} \rho \left(y,y\right)=\frac{1}{2\pi }\int \phi \left(1;\mu ,0\right)\exp\left(-i\mu y\right)d\mu =\frac{1}{\sqrt{2\pi }}\hat{\phi }\left(1;y,0\right), \end{array}$$

where $\hat{\phi }\left(1;y,0\right)$ is a Fourier transform of ϕ(1;μ,0). Since ρ(y,y)≥0 holds, Condition (23) follows.

Let (23) be valid. By the definition of the Fourier transformation, we can write

(25) $$\begin{array}{c} \hat{\phi }\left(1;y,0\right)=\frac{1}{\sqrt{2\pi }}\int \phi \left(1;\mu ,0\right)\exp\left(-i\mu y\right)d\mu . \end{array}$$

We compare this with the expression for $\rho (y,y^{\prime })$ from the previous proof for $y=y^{\prime }$ and conclude that it is the diagonal element of the density matrix. Since the left-hand side of the integral is nonnegative for any *y*, all the diagonal elements of the density matrix are also nonnegative. □

**Lemma** **3.** *(Unit trace) The integral in (9) defines the normalised operator $\hat{\rho }$ if and only if the characteristic function satisfies the condition*

(26) $$\begin{array}{c} \phi (1;0,0)=1. \end{array}$$

**Proof.**  The proof is straightforward from (10). □

We can summarise the latter results in the following theorem.

**Theorem** **1.** *The integral in (9), from the characteristic function, defines the density operator $\hat{\rho }$ corresponding to a quantum state if and only if the characteristic function satisfies Lemmas 1–3.*

**Corollary** **1.** *Since the purity parameter satisfies $0\leq Tr{\hat{\rho }}^{2}\leq 1$, the following condition:*

(27) $$\begin{array}{c} 0\leq \frac{1}{2\pi }\overset{\infty }{\underset{-\infty }{\;\int \int \;}}\phi \left(1;\mu ,\nu \right)\phi \left(1;-\mu ,-\nu \right)d\mu d\nu \leq 1, \end{array}$$

*holds. For two density matrices ${\hat{\rho }}_{1}$, ${\hat{\rho }}_{2}$, the trace overlap is $0\leq Tr\left({\hat{\rho }}_{1}{\hat{\rho }}_{2}\right)\leq 1$. Then, the following condition:*

(28) $$\begin{array}{c} 0\leq \frac{1}{2\pi }\overset{\infty }{\underset{-\infty }{\;\int \int \;}}\phi_{1}\left(1;\mu ,\nu \right)\phi_{2}\left(1;-\mu ,-\nu \right)d\mu d\nu \leq 1, \end{array}$$

*holds, where $\phi_{1}\left(\cdot \right)$, $\phi_{2}\left(\cdot \right)$ are the corresponding characteristic functions.*

Hence, if we want to reconstruct a state from a given tomographic pdf, we need to ensure that its characteristic function satisfies the Theorem 1. Next, we consider several examples.

### Harmonic Oscillator

The ground state of the harmonic oscillator (HO) is defined by the wave function

(29) $$\begin{array}{c} \Psi_{0}\left(y\right)=\pi^{-1/4}\exp\left(-y^{2}/2\right). \end{array}$$

The corresponding tomogram can be easily calculated:

(30) $$\begin{array}{c} W_{0}\left(X|\mu ,\nu \right)=\frac{1}{\sqrt{\pi (\mu^{2}+\nu^{2})}}\exp\left[-\frac{X^{2}}{\mu^{2}+\nu^{2}}\right]. \end{array}$$

The latter is a Gaussian pdf $N(0,\frac{\mu^{2}+\nu^{2}}{2})$. The corresponding characteristic function is

(31) $$\begin{array}{c} \phi_{0}\left(1;\mu ,\nu \right)=\exp\left[-\frac{\mu^{2}+\nu^{2}}{4}\right], \end{array}$$

and it is resistant to the change in the signs of μ, ν of the parameters. It is easy to check that Theorem 1 is fulfilled.

The wave function of the excited state of the harmonic oscillator is

(32) $$\begin{array}{c} \Psi_{n}\left(y\right)=\frac{1}{\sqrt[{4}]{\pi }\sqrt{2^{n}n!}}e^{-y^{2}/2}H_{n}\left(y\right), \end{array}$$

where $H_{n}\left(y\right)$ is the Hermite polynomial. We use the integral

(33) $$\begin{array}{c} \int_{-\infty }^{\infty }e^{-p{\left(x-y\right)}^{2}}H_{n}\left(cx\right)dx=\frac{\sqrt{\pi }{\left(p-c^{2}\right)}^{n/2}}{p^{(n+1)/2}}H_{n}\left(cy\sqrt{\frac{p}{p-c^{2}}}\right) \end{array}$$

to find the tomogram of the excited oscillator state:

(34) $$\begin{array}{c} W_{n}\left(X|\mu ,\nu \right)=W_{0}\left(X|\mu ,\nu \right)\frac{1}{2^{n}n!}{\left(H_{n}\left(\frac{X}{\sqrt{\mu^{2}+\nu^{2}}}\right)\right)}^{2}. \end{array}$$

To our best knowledge, the latter pdf does not arise in classical probability theory in any context except quantum mechanics. In some rare sources, it is named the Hermite–Gaussian pdf. We use the following integral:

(35) $$\begin{array}{c} \int_{-\infty }^{\infty }e^{-z^{2}+bz}H_{n}\left(z\right)H_{m}\left(z\right)dz=\sqrt{\pi }e^{\frac{b^{2}}{4}}n!m!\sum_{k=0}^{\min\left(n,m\right)}\frac{2^{k}b^{n+m-2k}}{k!(n-k)!(m-k)!}, \end{array}$$

to find its characteristic function

(36) $$\begin{array}{ccc} \phi_{n}\left(1;\mu ,\nu \right) & = & e^{\frac{-(\mu^{2}+\nu^{2})}{4}}L_{n}\left(\frac{\mu^{2}+\nu^{2}}{2}\right), \end{array}$$

where we used the Laguerre polynomial series decomposition

(37) $$\begin{array}{c} L_{n}\left(x\right)=\sum_{k=0}^{n}\frac{{\left(-1\right)}^{k}n!}{{\left(k!\right)}^{2}\left(n-k\right)!}x^{k}. \end{array}$$

One can check that the Theorem 1 is fulfilled.

## 4. Exponential Family of pdfs

As we can see from the example of harmonic oscillators, the pdfs in (30) and (34) can be written in the following form:

(38) $$\begin{array}{c} W\left(X|\eta \right)\equiv h\left(X\right)e^{\eta^{T}\tau \left(X\right)-A\left(\eta \right)}, \end{array}$$

which is a general expression for an exponential family of pdfs for a given vector of sufficient statistics τ(X) and natural parameters η. For example, for the ground state of the harmonic oscillator, the functions are the following: $\tau \left(X\right)=-X^{2}$, $\eta =-1/(\mu^{2}+\nu^{2})$, $h\left(X\right)=1/\sqrt{\pi }$. From the normalisation condition, we can find the cumulant generating function

(39) $$\begin{array}{c} e^{A(\eta )}=\int h\left(X\right)e^{\eta^{T}\tau \left(X\right)}dX. \end{array}$$

This shows that A(η) (often called “log normalizer”) is not a degree of freedom in the specification of an exponential family density. It is determined once η, τ(X), and h(X) are determined:

(40) $$\begin{array}{c} A\left(\eta \right)=\log\left(\int h\left(X\right)e^{\eta^{T}\tau \left(X\right)}dX\right). \end{array}$$

The characteristic function (7) at the point t=1 for such a family of pdfs can be written as follows:

(41) $$\begin{array}{c} \phi \left(1;\eta \left(\mu ,\nu \right)\right)=\int e^{iX}W\left(X|\eta \right)dX=e^{-A(\eta (\mu ,\nu ))}\int e^{iX+\eta {\left(\mu ,\nu \right)}^{T}\tau \left(X\right)}h\left(X\right)dX, \end{array}$$

where η(μ,ν) is a vector that depends on μ, ν. If one wants to check the known pdf from the exponential family to generate the quantum state, one needs to check that the latter characteristic function satisfies Theorem 1.

Hence, any pdf from the exponential family can be checked on the latter conditions. Further, we provide more detailed analyses for the most important pdfs known in probability theory.

### Special Cases

Let us observe τ(X)=X, $X\in R^{+}$; A(η) is the log of Laplace transform of h(X). The Laplace transform of h(X) is defined as follows:

(42) $$\begin{array}{c} H\left(s\right)=\int_{0}^{\infty }h\left(X\right)e^{-sX}dX, \end{array}$$

where *s* is a complex frequency-domain parameter s=σ+iω, with σ,ω∈R. A necessary condition for existence of the integral is that h(X) must be locally integrable on (0,∞) and η<0. Then, the characteristic function at t=1 is

(43) $$\begin{array}{c} \phi \left(1;\eta \right)={\left(\int_{0}^{\infty }h\left(X_{1}\right)e^{\eta X_{1}}dX_{1}\right)}^{-1}\left(\int_{0}^{\infty }h\left(X_{2}\right)e^{\left(i+\eta \right)X_{2}}dX_{2}\right). \end{array}$$

**Example** **1.** *Let us observe $h\left(X\right)=X^{\alpha -1}$, α>0, and η=−p(μ,ν), where p(μ,ν) is a nonnegative function. Then, the pdf is*

(44) $$\begin{array}{c} f\left(X|\mu ,\nu \right)=X^{\alpha -1}e^{-p(\mu ,\nu )X-A(p(\mu ,\nu ))},\;X\in R^{+}. \end{array}$$

*Then, the characteristic function is*

(45) $$\begin{array}{c} \phi \left(1;\mu ,\nu \right)=\frac{p^{\alpha }\left(\mu ,\nu \right)}{{\left(p\left(\mu ,\nu \right)-i\right)}^{\alpha }}. \end{array}$$

*Lemma 1 is fulfilled if $p\left(\mu ,\nu \right)=-p^{★}\left(\mu ,-\nu \right)$. We can write the function p(μ,ν) as*

(46) $$\begin{array}{c} p(\mu ,\nu )=A(\mu ,\nu )+B(\mu ,\nu )i, \end{array}$$

*where A(μ,ν), B(μ,ν) are real functions. Then, one can write the conditions*

(47) $$\begin{array}{c} A(\mu ,\nu )=-A(\mu ,-\nu ),\;B(\mu ,\nu )=B(\mu ,-\nu ). \end{array}$$

*As an example, one can select A(μ,ν)=sinμsinν, B(μ,ν)=sinμcosν. For Lemma 2, we need the Fourier transform of the characteristic function in ν=0 to be a real-valued, nonnegative function. This implies the condition Re(p(μ,0))=0, i.e., A(μ,0)=0. Our example function fulfills this condition. Lemma 2 is satisfied if $F\left(\frac{B^{\alpha }\left(y,0\right)}{{\left(B\left(y,0\right)-1\right)}^{\alpha }}\right)\geq 0$, ∀y, where F(·) denotes the Fourier transform. Note that the function being nonnegative does not imply that its transform is also nonnegative. The condition of the Lemma 3 is not satisfied since one cannot find p(0,0) such that ϕ(1;0,0)=1. Hence, this class of pdfs is not suitable for a tomogram. This class includes important pdfs like exponential, gamma, and $\chi^{2}$ pdfs, showing that these do not satisfy the necessary conditions.*

Let us observe $\tau \left(X\right)={\left(X,X^{2}\right)}^{T}$, $\eta =(\eta_{1},\eta_{2})$, X∈R. Then, the characteristic function is

(48) $$\begin{array}{c} \phi \left(1;\eta \right)={\left(\int_{-\infty }^{\infty }h\left(X_{1}\right)e^{\eta_{1}X_{1}+\eta_{2}X_{1}^{2}}dX_{1}\right)}^{-1}\left(\int_{-\infty }^{\infty }h\left(X_{2}\right)e^{\left(i+\eta_{1}\right)X_{2}+\eta_{2}X_{2}^{2}}dX_{2}\right). \end{array}$$

**Example** **2.** *For a special case of h(X)=C, where C is a constant and $\eta_{1}=p_{1}\left(\mu ,\nu \right)$, $\eta_{2}=-p_{2}\left(\mu ,\nu \right)$, where $p_{i}\left(\mu ,\nu \right)$, i=1,2 are nonnegative functions, the characteristic function is the following:*

(49) $$\begin{array}{c} \phi \left(1;p\left(\mu ,\nu \right)\right)=e^{-\frac{2ip_{1}\left(\mu ,\nu \right)-1}{4p_{2}\left(\mu ,\nu \right)}}. \end{array}$$

*If we take $p_{1}\left(\mu ,\nu \right)=0$, $p_{2}\left(\mu ,\nu \right)=-1/\left(\mu^{2}+\nu^{2}\right)$, the case corresponds to harmonic oscillator (31) and the tomogram is the Gaussian distribution.*

## 5. Pseudoharmonic Oscillator

The straight motion along the positive *x*-semi-axis can be described by potential

(50) $$\begin{array}{c} V^{1D}\left(a,x\right)=D_{\omega }{\left(\frac{x}{x_{\omega }}-\sqrt{a}\frac{x_{\omega }}{x}\right)}^{2},\;0\leq x\leq \infty , \end{array}$$

where the dimensionless coefficient a≥0, $D_{\omega }=\frac{\hbar \omega }{2}$, ω is the confining frequency, and $x_{\omega }=\frac{\hbar }{m\omega }$. At a=0, Equation (50) describes the geometry of a particle confined to the right-hand half of the harmonic oscillator of the frequency ω. Problem (50) is cited in the literature as pseudoharmonic oscillator (PHO) [34]. The 1D Schrödinger equation is

(51) $$\begin{array}{c} \frac{\hbar^{2}}{2m}\frac{d^{2}}{dx^{2}}\Psi_{n}\left(a;x\right)+V^{1D}\left(a,x\right)\Psi_{n}\left(a;x\right)=E_{n}\left(a\right)\Psi_{n}\left(a;x\right). \end{array}$$

For the *a*-dependent potential (50), the energies and the wave function are

(52) $$\begin{array}{ccc} & & E_{n}\left(a\right)=\hbar \omega \left(2n+1+b-\sqrt{a}\right),\\ & & \Psi_{n}\left(a;x\right)=\frac{1}{\sqrt{x_{\omega }}}{\left[\frac{2n!}{\Gamma (n+b+1)}\right]}^{\frac{1}{2}}{\left(\frac{x}{x_{\omega }}\right)}^{b+\frac{1}{2}}\exp\left(-\frac{1}{2}\frac{x^{2}}{x_{\omega }^{2}}\right)L_{n}^{\left(\eta \right)}\left(\frac{x^{2}}{x_{\omega }^{2}}\right), \end{array}$$

where $b=\frac{1}{2}\sqrt{1+4a}$, Γ(z) is a Γ-function and $L_{n}^{\left(b\right)}$ is an *n*th-order associated Laguerre polynomial. The divergence of the potential at the left edge x=0 forces the function to vanish there at any *a* and *n*: $\Psi_{n}\left(a;0\right)=0$. At a=0, the spatial dependence reads

(53) $$\begin{array}{ccc} & & \Psi_{n}\left(0;x\right)=\frac{{\left(-1\right)}^{n}}{x_{\omega }^{\frac{1}{2}}}\frac{1}{2^{2n+\frac{1}{2}}}{\left[\frac{1}{n!\Gamma (n+\frac{3}{2})}\right]}^{\frac{1}{2}}\exp\left(-\frac{1}{2}\frac{x^{2}}{x_{\omega }^{2}}\right)H_{2n+1}\left(\frac{x}{x_{\omega }}\right). \end{array}$$

Then, one can find the tomogram $W_{n}\left(X|a,\mu ,\nu \right)$ of (53):

(54) $$\begin{array}{ccc} W_{n}\left(X|0,\mu ,\nu \right)\;\; & = & \;\;\frac{1}{2\pi |\nu |}\frac{1}{x_{\omega }}\frac{1}{2^{4n+1}}\frac{1}{n!\Gamma (n+\frac{3}{2})}I\left(X\right),\\ I(X) & \equiv & |\int_{0}^{\infty }\exp\left[-x^{2}\left(\frac{1}{2x_{\omega }^{2}}-\frac{i\mu }{2\nu }\right)-\frac{iX}{\nu }x\right]H_{2n+1}\left(\frac{x}{x_{\omega }}\right)dx{|}^{2}. \end{array}$$

The closed form of the latter tomogram is derived in Appendix B:

(55) $$\begin{array}{ccc} & & W_{n}\left(X|0,\mu ,\nu \right)\;\;=\;\;\frac{1}{\pi }\frac{{\left(\left(2n+1\right)!\right)}^{2}}{n!(n+\frac{1}{2})!}\frac{\nu^{2n+1}x_{\omega }}{{\left(\nu^{2}+\mu^{2}x_{\omega }^{4}\right)}^{n+1}}e^{-\frac{X^{2}x_{\omega }^{2}}{2\left(\nu^{2}+\mu^{2}x_{\omega }^{4}\right)}}\\ & \times & [\sum_{m_{1},m_{2}=0}^{n}\frac{{\left(-1\right)}^{m_{1}+m_{2}}}{m_{1}!m_{2}!}{\left(\frac{x_{\omega }^{2}}{16\nu }\right)}^{m_{1}+m_{2}}{\left(\nu -i\mu x_{\omega }^{2}\right)}^{m_{1}}{\left(\nu +i\mu x_{\omega }^{2}\right)}^{m_{2}}\\ & \times & D_{-(2n-2m_{1}+2)}\left(\frac{iXx_{\omega }\sqrt{\nu +i\mu x_{\omega }^{2}}}{\sqrt{\nu }\sqrt{\nu^{2}+\mu^{2}x_{\omega }^{4}}}\right)D_{-(2n-2m_{2}+2)}\left(\frac{iXx_{\omega }\sqrt{\nu -i\mu x_{\omega }^{2}}}{\sqrt{\nu }\sqrt{\nu^{2}+\mu^{2}x_{\omega }^{4}}}\right)]. \end{array}$$

**Figure 1:**
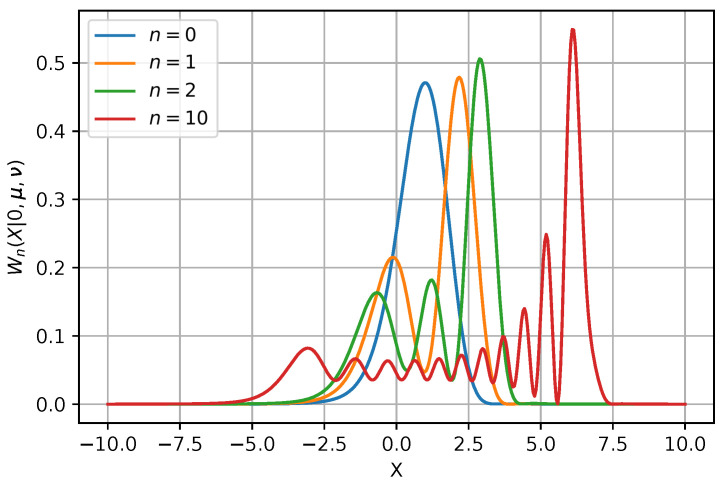
The tomogram $W_{n}\left(X|0,\mu ,\nu \right)$ for the PHO (53) is plotted for n∈{0,1,2,10}. The ground-state tomogram is a Gaussian pdf with the shifted center, while the excited states are the multi-mode pdfs from the exponential family.

This pdf is a so-called normal–exponential–gamma distribution type. It is depicted in Figure 1 for the excitations n={0,1,2,10}. The distribution is not symmetrical, with the center of the pdf shifted to the right side of the *X*-axis, whereas the pdf of a standard harmonic oscillator is centered at X=0. The characteristic function of the latter distribution, defined by Transformation (8), is quite cumbersome. We have numerically verified that it satisfies the conditions in Theorem 1.

Next, we move to the tomogram for the general case (53). According to the definition, it can be written as

(56) $$\begin{array}{c} W\left(X|a,\mu ,\nu \right)=\frac{1}{2\pi |\nu |}\frac{1}{x_{\omega }}\left[\frac{2n!}{\Gamma (n+b+1)}\right]|\int_{0}^{\infty }{\left(\frac{y}{x_{\omega }}\right)}^{b+\frac{1}{2}}e^{\left(-\frac{1}{2}\frac{y^{2}}{x_{\omega }^{2}}\right)}L_{n}^{\left(b\right)}\left(\frac{y^{2}}{x_{\omega }^{2}}\right)e^{\left(\frac{i\mu }{2\nu }y^{2}-\frac{iX}{\nu }y\right)}dy{|}^{2}. \end{array}$$

The closed form is

(57) $$\begin{array}{ccc} & & W_{n}\left(X|a,\mu ,\nu \right)=\frac{x_{\omega }}{2\pi |\nu |}\left[\frac{2n!}{\Gamma (n+b+1)}\right]\exp\left(-\frac{X^{2}x_{\omega }^{2}}{2(\nu^{2}+\mu^{2}x_{\omega }^{4})}\right){\left(\frac{{\left(b+1\right)}_{n}}{n}\right)}^{2}\\ & \times & |\sum_{k=0}^{\infty }\frac{{\left(n\right)}_{k}}{{\left(b+1\right)}_{k}k!}\Gamma \left(2k+b+\frac{3}{2}\right){\left(\frac{\nu -i\mu x_{\omega }^{2}}{\nu }\right)}^{-(k+\frac{b}{2}+\frac{3}{4})}D_{-(2k+b+\frac{3}{2})}\left(\frac{iXx_{\omega }\sqrt{\nu +i\mu x_{\omega }^{2}}}{\sqrt{\nu }\sqrt{\nu^{2}+\mu^{2}x_{\omega }^{4}}}\right){|}^{2}. \end{array}$$

**Figure 2:**
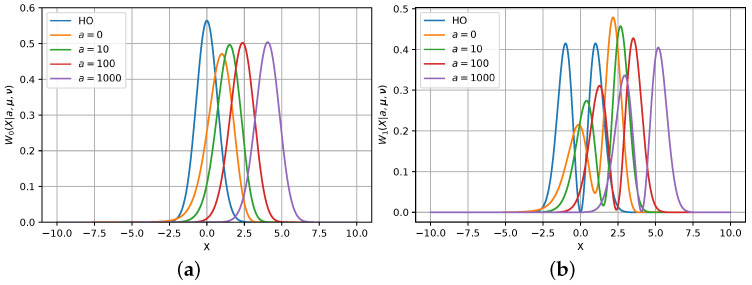
Comparison of the tomograms of HO and PHO for a∈{0,10,100,1000}: (**a**) The ground-state (n=0) tomograms are the Gaussian pdfs, where, for the PHO, the center of the pdf is shifted; (**b**) the first excited-state (n=1) tomograms are from the exponential family of pdfs with multiple modes.

Using the latter representation, we can examine the dependence of $W_{n}\left(X|a,\mu ,\nu \right)$ from *a* and compare it to the $W_{n}\left(X|\mu ,\nu \right)$ pdf. Both functions are depicted in Figure 2 for n={0,1}. We can observe that the larger the value of *a* becomes, the further the function $W_{n}\left(X|a,\mu ,\nu \right)$ is shifted to the right. The shape of the pdf changes slightly with the growth of *a*. However, for the first exited state, the mass of the picks of the distribution is slightly changing.

We observe that, for both HO and PHO, the tomograms deviate from Gaussian behavior, even for the first excited state. The distribution becomes multi-modal and challenging to estimate. Subsequently, we examine the case of an even more complex distribution arising in superposition states.

## 6. Crystallised Cat States

The even and odd coherent states are introduced in [44] from the Glauber coherent states |α〉 of the harmonic oscillator as

(58) $$\begin{array}{c} |\alpha_{\pm }\rangle =N_{\pm }\left(|\alpha \rangle \pm |-\alpha \rangle \right),\;N_{\pm }={\left(2(1\pm e^{-{2|\alpha |}^{2}})\right)}^{-1/2}. \end{array}$$

Their generalisation is provided in [44], using the Abelian symmetry group $C_{3}$ with three rotation elements $\left\{1,e^{2\pi i/3},e^{4\pi i/3}\right\}$ acting on the coherent states, giving the following state:

(59) $$\begin{array}{c} |\psi \rangle =N\sum_{j=1}^{3}|\psi_{j}\rangle ,\;|\psi_{1}\rangle =|\alpha \rangle ,\;|\psi_{2}\rangle =|\alpha e^{2\pi i/3}\rangle ,\;|\psi_{3}\rangle =|\alpha e^{4\pi i/3}\rangle . \end{array}$$

The coordinate representation of the coherent state reads as

(60) $$\begin{array}{c} \psi_{\alpha }\left(x\right)=\langle x|\alpha \rangle =\pi^{-1/4}\exp\left(-\frac{x^{2}}{2}-\frac{{\left|\alpha \right|}^{2}}{2}+\sqrt{2}\alpha x-\frac{\alpha^{2}}{2}\right), \end{array}$$

and its tomogram is known to be equal to [26]

(61) $$\begin{array}{c} W\left(X|\alpha ,\mu ,\nu \right)=\frac{e^{-{\left|\alpha \right|}^{2}}e^{\frac{{\left(\nu +i\mu \right)}^{2}\alpha^{2}+{\left(\nu -i\mu \right)}^{2}{\left(\alpha^{★}\right)}^{2}}{2(\nu^{2}+\mu^{2})}}}{\sqrt{\pi (\nu^{2}+\mu^{2})}}e^{-\frac{X^{2}}{\nu^{2}+\mu^{2}}}e^{\frac{\sqrt{2}iX\left(\left(\nu -i\mu \right)\alpha^{★}-\left(\nu +i\mu \right)\alpha \right)}{\nu^{2}+\mu^{2}}}. \end{array}$$

The characteristic function is

(62) $$\begin{array}{c} \phi \left(1;\alpha ,\mu ,\nu \right)=e^{-\frac{\nu^{2}+\mu^{2}}{4}}e^{-\frac{\left(\nu -i\mu \right)\alpha^{★}-\left(\nu +i\mu \right)\alpha }{\sqrt{2}}}. \end{array}$$

We can write every state in (59) as

(63) $$\begin{array}{ccc} & & \psi_{j}\left(x\right)=N_{j}\exp\left(-A_{j}x^{2}+B_{j}x+C_{j}\right),\;j=1,2,3,\\ & & A_{j}=\frac{1}{2},\;B_{j}=\sqrt{2}\alpha e^{\frac{2\pi i}{3}\left(j-1\right)},\;C_{j}=-\frac{{\left|\alpha \right|}^{2}}{2}-\frac{\alpha^{2}e^{\frac{4\pi i}{3}\left(j-1\right)}}{2},\;N_{j}=\pi^{-1/4}. \end{array}$$

According to (6), the tomogram can be written as

(64) $$\begin{array}{ccc} W_{ccat}\left(X|\alpha ,\mu ,\nu \right) & = & \frac{{\left|N\right|}^{2}e^{-{\left|\alpha \right|}^{2}}}{\sqrt{\pi (\nu^{2}+\mu^{2})}}e^{-\frac{X^{2}}{\nu^{2}+\mu^{2}}}\\ & \times & \sum_{j,k=1}^{3}\exp\left(\frac{\sqrt{2}iX\left(\left(\nu -i\mu \right)\alpha^{★}e^{-\frac{2\pi i}{3}\left(k-1\right)}-\left(\nu +i\mu \right)\alpha e^{\frac{2\pi i}{3}\left(j-1\right)}\right)}{\nu^{2}+\mu^{2}}\right)\\ & \times & \exp\left(\frac{\left({\left(\nu +i\mu \right)}^{2}\alpha^{2}e^{\frac{4\pi i}{3}\left(j-1\right)}+{\left(\nu -i\mu \right)}^{2}{\left(\alpha^{★}\right)}^{2}e^{-\frac{4\pi i}{3}\left(k-1\right)}\right)}{2(\nu^{2}+\mu^{2})}\right). \end{array}$$

One can see that, if k,j=1, the tomogram coincides with the single coherent-state tomogram (61). The pdf in (64) is a mixture of the Gaussian distributions [45]. Note that the sum of Gaussian pdfs in general is not a Gaussian pdf itself.

The characteristic function is the following:

(65) $$\begin{array}{ccc} \phi_{ccat}\left(1;\alpha ,\mu ,\nu \right) & = & {\left|N\right|}^{2}e^{-\frac{\nu^{2}+\mu^{2}}{4}}\sum_{j,k=1}^{3}e^{-{\left|\alpha \right|}^{2}\left(\exp(-\frac{2\pi i}{3}\left(k-j\right)+1\right)}\\ & \times & e^{-\frac{\left(\nu -i\mu \right)\alpha^{★}\exp\left(-\frac{2\pi i}{3}\left(k-1\right)\right)-\left(\nu +i\mu \right)\alpha \exp\left(\frac{2\pi i}{3}\left(j-1\right)\right)}{\sqrt{2}}}. \end{array}$$

One can check that this satisfies Theorem 1.

## 7. Summary

For the accurate reconstruction of a quantum state, various techniques are employed. The tomographic pdf, as the true distribution function, completely characterises the state and has potential to be a convenient method for quantum-state reconstruction. When applying classical probability theory methods for estimating the pdf, it is often necessary to know which distribution family we are working with. In this article, we thoroughly investigated the conditions (Theorem 1) that the characteristic function of the distribution must satisfy to describe a physical system. This characteristic function is obtained as the Fourier transform of the pdf tomogram, aiming to describe a physical quantum state. These conditions strictly limit the family of pdfs that are suitable for describing quantum states. We specifically explored the exponential family of densities, introducing conditions that the characteristic function of a quantum state from this family must satisfy. We tested several well-known distributions, such as exponential, gamma, and $\chi^{2}$, demonstrating their unsuitability for generating quantum states.

Using the characteristic function, it is possible to express key features of a quantum state, e.g., the purity parameter. Additionally, we introduced a trace distance between two states based on their characteristic functions. Consequently, knowledge of the estimate of the characteristic function can serve as a test for the purity or proximity of two quantum states.

We investigated well-known states such as the quantum oscillator (symmetric and asymmetric), coherent states, cat states, and their superpositions. The tomograms of these states are associated with Gaussian and Hermite–Gaussian distributions, or mixtures of these distributions, as shown in Table 1. Their characteristic functions satisfy Theorem 1, as expected.

The study clearly demonstrates that the family of densities that are capable of generating quantum states is extremely limited. Further development of the article could involve a detailed examination of well-known density distributions, exploring their potential as tomograms for quantum states. Our intuition suggests that the presence of a quadratic exponential term (as in the tomography of the oscillator and all other tomograms in Table 1) is likely mandatory. It would be particularly interesting to investigate the power-law family of distributions, which finds extensive application in classical mathematics too. This study provides insights into the family of distributions encountered in quantum mechanics. With this information, one can employ both parametric and nonparametric methods to estimate the tomograms of quantum states. This investigation will be the focus of our future work.

## Figures and Tables

**Table 1:** Tomograms and characteristic functions for the ground and excited states of the oscillator and pseudoharmonic oscillator, as well as for coherent states and superpositions of cat states.

Quantum State	Tomogram (pdf) Characteristic Function
HO (ground state)	$W_{0}\left(X|\mu ,\nu \right)=\frac{1}{\sqrt{\pi (\mu^{2}+\nu^{2})}}\exp\left[-\frac{X^{2}}{\mu^{2}+\nu^{2}}\right]$,
	$\phi_{0}\left(1|\mu ,\nu \right)=\exp\left[-\frac{\mu^{2}+\nu^{2}}{4}\right]$
HO (excited state)	$W_{n}\left(X|\mu ,\nu \right)=W_{0}\left(X|\mu ,\nu \right)\frac{1}{2^{n}n!}{\left(H_{n}\left(\frac{X}{\sqrt{\mu^{2}+\nu^{2}}}\right)\right)}^{2}$
	$\phi_{n}\left(1;\mu ,\nu \right)=\phi_{0}\left(1|\mu ,\nu \right)L_{n}\left(\frac{\mu^{2}+\nu^{2}}{2}\right)$
PHO (a=0)	$W_{n}\left(X|\frac{1}{2},\mu ,\nu \right)\;\;=\;\;\frac{1}{\pi }\frac{{\left(\left(2n+1\right)!\right)}^{2}}{n!(n+\frac{1}{2})!}\frac{\nu^{2n+1}x_{\omega }}{{\left(\nu^{2}+\mu^{2}x_{\omega }^{4}\right)}^{n+1}}e^{-\frac{X^{2}x_{\omega }^{2}}{2\left(\nu^{2}+\mu^{2}x_{\omega }^{4}\right)}}$
	$\times [\sum_{m_{1},m_{2}=0}^{n}\frac{{\left(-1\right)}^{m_{1}+m_{2}}}{m_{1}!m_{2}!}{\left(\frac{x_{\omega }^{2}}{16\nu }\right)}^{m_{1}+m_{2}}{\left(\nu -i\mu x_{\omega }^{2}\right)}^{m_{1}}{\left(\nu +i\mu x_{\omega }^{2}\right)}^{m_{2}}$
	$\times D_{-(2n-2m_{1}+2)}\left(\frac{iXx_{\omega }\sqrt{\nu +i\mu x_{\omega }^{2}}}{\sqrt{\nu }\sqrt{\nu^{2}+\mu^{2}x_{\omega }^{4}}}\right)D_{-(2n-2m_{2}+2)}\left(\frac{iXx_{\omega }\sqrt{\nu -i\mu x_{\omega }^{2}}}{\sqrt{\nu }\sqrt{\nu^{2}+\mu^{2}x_{\omega }^{4}}}\right)].$
PHO (∀a)	$W_{n}\left(X|\eta ,\mu ,\nu \right)=\frac{x_{\omega }}{2\pi |\nu |}\left[\frac{2n!}{\Gamma (n+\eta +1)}\right]\exp\left(-\frac{X^{2}x_{\omega }^{2}}{2(\nu^{2}+\mu^{2}x_{\omega }^{4})}\right){\left(\frac{{\left(\eta +1\right)}_{n}}{n}\right)}^{2}$
	$|\sum_{k=0}^{\infty }\frac{{\left(n\right)}_{k}}{{\left(\eta +1\right)}_{k}k!}\Gamma \left(2k+\eta +\frac{3}{2}\right){\left(\frac{\nu -i\mu x_{\omega }^{2}}{\nu }\right)}^{-(k+\frac{\eta }{2}+\frac{3}{4})}\;\;\;D_{-(2k+\eta +\frac{3}{2})}\left(\frac{iXx_{\omega }\sqrt{\nu +i\mu x_{\omega }^{2}}}{\sqrt{\nu }\sqrt{\nu^{2}+\mu^{2}x_{\omega }^{4}}}\right){|}^{2}$
Coherent state	$W\left(X|\alpha ,\mu ,\nu \right)=W_{0}\left(X|\mu ,\nu \right)e^{-{\left|\alpha \right|}^{2}}e^{\frac{{\left(\nu +i\mu \right)}^{2}\alpha^{2}+{\left(\nu -i\mu \right)}^{2}{\left(\alpha^{★}\right)}^{2}}{2(\nu^{2}+\mu^{2})}}e^{\frac{\sqrt{2}iX\left(\left(\nu -i\mu \right)\alpha^{★}-\left(\nu +i\mu \right)\alpha \right)}{\nu^{2}+\mu^{2}}}$
	$\phi \left(1;\alpha ,\mu ,\nu \right)=\phi_{0}\left(1;\mu ,\nu \right)e^{-\frac{\left(\nu -i\mu \right)\alpha^{★}-\left(\nu +i\mu \right)\alpha }{\sqrt{2}}}$
Crystallised cat states	$W_{ccat}\left(X|\alpha ,\mu ,\nu \right)=W_{0}\left(X|\mu ,\nu \right)e^{-{\left|\alpha \right|}^{2}}{\left|N\right|}^{2}$
	$\;\;\times \;\;\sum_{j,k=1}^{3}e^{\frac{{\left(\nu +i\mu \right)}^{2}\alpha^{2}e^{\frac{4\pi i}{3}\left(j-1\right)}+{\left(\nu -i\mu \right)}^{2}{\left(\alpha^{★}\right)}^{2}e^{-\frac{4\pi i}{3}\left(k-1\right)}}{2(\nu^{2}+\mu^{2})}}\;\;e^{\frac{\sqrt{2}iX\left(\left(\nu -i\mu \right)\alpha^{★}e^{-\frac{2\pi i}{3}\left(k-1\right)}-\left(\nu +i\mu \right)\alpha e^{\frac{2\pi i}{3}\left(j-1\right)}\right)}{\nu^{2}+\mu^{2}}}$
	$\phi_{ccat}\left(1;\alpha ,\mu ,\nu \right)={\left|N\right|}^{2}\phi_{0}\left(1;\mu ,\nu \right)$
	$\sum_{j,k=1}^{3}e^{-{\left|\alpha \right|}^{2}\left(\exp(-\frac{2\pi i}{3}\left(k-j\right)+1\right)}e^{-\frac{\left(\nu -i\mu \right)\alpha^{★}\exp\left(-\frac{2\pi i}{3}\left(k-1\right)\right)-\left(\nu +i\mu \right)\alpha \exp\left(\frac{2\pi i}{3}\left(j-1\right)\right)}{\sqrt{2}}}$

## Data Availability

All necessary data are contained in the article.

## Appendix A. Matrix Elements of the Density Matrix

To write the condition $\rho \left(y,y^{\prime }\right)=\rho {\left(y^{\prime },y\right)}^{★}$ in terms of the characteristic function, we use the Baker–Campbell–Hausdorff formula $e^{ia\hat{q}}e^{ib\hat{p}}e^{\frac{iab\hbar }{2}}=e^{i\left(a\hat{q}+b\hat{p}\right)}$. We obtain

(A1) $$\begin{array}{c} \exp\left(i\left(X\hat{1}-\mu \hat{q}-\nu \hat{p}\right)\right)=\exp\left(iX\right)\exp\left(-i\mu \hat{q}\right)\exp\left(-i\nu \hat{p}\right)\exp\left(i\frac{\mu \nu }{2}\right). \end{array}$$

The matrix element of the latter is

(A2) $$\begin{array}{ccc} {\left[\exp\left(i\left(X\hat{1}-\mu \hat{q}-\nu \hat{p}\right)\right)\right]}_{yy^{\prime }} & = & \exp\left(iX\right)\exp\left(i\frac{\mu \nu }{2}\right){\left[\exp\left(-i\mu \hat{q}\right)\exp\left(-i\nu \hat{p}\right)\right]}_{yy^{\prime }}\\ & = & \exp\left(iX\right)\exp\left(i\frac{\mu \nu }{2}\right){\left[\exp\left(-i\mu \hat{q}\right)\right]}_{yx}{\left[\exp\left(-i\nu \hat{p}\right)\right]}_{xy^{\prime }}. \end{array}$$

The matrix element from the latter expression is

(A3) $$\begin{array}{c} {\left[\exp\left(-i\mu \hat{q}\right)\right]}_{yx}=\langle y|\exp\left(-i\mu \hat{q}\right)|x\rangle =\exp\left(-i\mu x\right)\delta \left(y-x\right). \end{array}$$

The translation operator $\hat{T}\left(\nu \right)=\exp\left(-i\nu \hat{p}/\hbar \right)$ moves particles and fields by the amount ν, namely,

(A4) $$\begin{array}{ccc} & & e^{-i\nu \hat{p}}\Psi \left(x\right)=\Psi \left(x+\nu \right),\;e^{-i\nu \hat{p}}|x\rangle =|x+\nu \rangle . \end{array}$$

Thus, we have

(A5) $$\begin{array}{c} {\left[\exp\left(-i\nu \hat{p}\right)\right]}_{xy^{\prime }}=\langle x|\exp\left(-i\nu \hat{p}\right)|y^{\prime }\rangle =\langle x|y^{\prime }+\nu \rangle =\delta \left(x-y^{\prime }-\nu \right). \end{array}$$

Using (5), we can write the matrix element of the density matrix as follows:

(A6) $$\begin{array}{c} \rho \left(y,y^{\prime }\right)=\frac{1}{2\pi }\int \phi \left(1;\mu ,y-y^{\prime }\right)\exp\left(-i\frac{\mu (y+y^{\prime })}{2}\right)d\mu . \end{array}$$

The conjugate transpose element is

(A7) $$\begin{array}{c} \rho^{★}\left(y^{\prime },y\right)=\frac{1}{2\pi }\int \phi \left(-1;\mu ,y^{\prime }-y\right)\exp\left(-i\frac{\mu (y+y^{\prime })}{2}\right)d\mu . \end{array}$$

## Appendix B. Tomogram for the Pseudoharmonic Oscillator with *A* = 0

Let us first find the tomogram $W_{n}\left(X|a,\mu ,\nu \right)$ of (53):

(A8) $$\begin{array}{ccc} W_{n}\left(X|0,\mu ,\nu \right)\;\; & = & \;\;\frac{1}{2\pi |\nu |}\frac{1}{x_{\omega }}\frac{1}{2^{4n+1}}\frac{1}{n!\Gamma (n+\frac{3}{2})}I\left(X\right),\\ I(X) & \equiv & |\int_{0}^{\infty }\exp\left[-x^{2}\left(\frac{1}{2x_{\omega }^{2}}-\frac{i\mu }{2\nu }\right)-\frac{iX}{\nu }x\right]H_{2n+1}\left(\frac{x}{x_{\omega }}\right)dx{|}^{2}. \end{array}$$

Using the series expression of the Hermitian polynomial:

(A9) $$\begin{array}{c} H_{2n+1}\left(\frac{x}{x_{\omega }}\right)=\left(2n+1\right)!\sum_{m=0}^{n}\frac{{\left(-1\right)}^{m}}{m!(2n-2m+1)!}{\left(\frac{2x}{x_{\omega }}\right)}^{2n-2m+1}, \end{array}$$

we can rewrite the integral in the tomogram as

(A10) $$\begin{array}{c} I\left(X\right)={\left(\left(2n+1\right)!\right)}^{2}|\sum_{m=0}^{n}\frac{{\left(-1\right)}^{m}}{m!(2n-2m+1)!}\int_{0}^{\infty }e^{\left[-x^{2}\left(\frac{1}{2x_{\omega }^{2}}-\frac{i\mu }{2\nu }\right)-\frac{iX}{\nu }x\right]}{\left(\frac{2x}{x_{\omega }}\right)}^{2n-2m+1}dx{|}^{2}. \end{array}$$

We use the integral

(A11) $$\begin{array}{ccc} & & \int_{0}^{\infty }x^{\alpha -1}e^{-px^{2}-qx}dx=\Gamma \left(\alpha \right){\left(2p\right)}^{-\alpha /2}\exp\left(q^{2}/8p\right)D_{-\alpha }\left(q/\sqrt{2p}\right),\\ & & [ℜ\left(\alpha \right),ℜ\left(p\right)>0],\;\mathrm{or}\;[ℜ\left(\alpha \right),ℜ\left(q\right)>0,ℜ\left(p\right)=0],\\ & & \mathrm{or}\;[0<ℜ\left(\alpha \right)<2,ℜ\left(q\right)=ℜ\left(p\right)=0,ℑ\left(p\right)\neq 0], \end{array}$$

where $D_{a}\left(z\right)$ are parabolic cylinder functions, to write

(A12) $$\begin{array}{ccc} I(X) & = & {\left(\left(2n+1\right)!\right)}^{2}e^{-\frac{X^{2}x_{\omega }^{2}}{2\left(\nu^{2}+\mu^{2}x_{\omega }^{4}\right)}}|\sum_{m=0}^{n}\frac{{\left(-1\right)}^{m}}{m!(2n-2m+1)!}\Gamma \left(2n-2m+2\right)\\ & \times & D_{-2n+2m-2}\left(\frac{iXx_{\omega }}{\sqrt{\nu }\sqrt{\nu -i\mu x_{\omega }^{2}}}\right){\left(\frac{2}{x_{\omega }}\right)}^{2n-2m+1}{\left(\frac{\nu -i\mu x_{\omega }^{2}}{\nu x_{\omega }^{2}}\right)}^{-n+m-1}{|}^{2}. \end{array}$$

Then, the tomogram is

(A13) $$\begin{array}{ccc} & & W_{n}\left(X|0,\mu ,\nu \right)\;\;=\;\;\frac{1}{\pi }\frac{{\left(\left(2n+1\right)!\right)}^{2}}{n!\Gamma (n+\frac{3}{2})}\frac{\nu^{2n+1}x_{\omega }}{{\left(\nu^{2}+\mu^{2}x_{\omega }^{4}\right)}^{n+1}}e^{-\frac{X^{2}x_{\omega }^{2}}{2\left(\nu^{2}+\mu^{2}x_{\omega }^{4}\right)}}\\ & \times & |\sum_{m=0}^{n}\frac{{\left(-1\right)}^{m}}{m!(2n-2m+1)!}\Gamma \left(2n-2m+2\right)\\ & \times & D_{-(2n-2m+2)}\left(\frac{iXx_{\omega }\sqrt{\nu +i\mu x_{\omega }^{2}}}{\sqrt{\nu }\sqrt{\nu^{2}+\mu^{2}x_{\omega }^{4}}}\right){\left(\frac{\left(\nu -i\mu x_{\omega }^{2}\right)}{4\nu }\right)}^{m}{\left(\frac{x_{\omega }}{2}\right)}^{2m}{|}^{2}. \end{array}$$

We use the series representation

(A14) Dν(z)=2−ν2e−z24Γ(12+ν)F11(−ν,12,12z2),

where F11(a,b,z) is the confluent hypergeometric function of the first kind. It is also known that

(A15) F11(a;b;z)=∑k=0∞(a)kzk(b)kk!,

where $a_{k}$ is the Pochhammer symbol: ${\left(a\right)}_{k}=a\left(a+1\right)\ldots \left(a+k-1\right)$, k=1,2,3…, ${\left(a\right)}_{0}=1$. Combining this knowledge, we can write

(A16) $$\begin{array}{c} D_{\nu }\left(z\right)=\frac{2^{-\frac{\nu }{2}}e^{-\frac{z^{2}}{4}}}{\Gamma (\frac{1}{2}+\nu )}\sum_{k=0}^{\infty }\frac{{\left(-\nu \right)}_{k}z^{2k}}{2^{k}{\left(\frac{1}{2}\right)}_{k}k!}=\frac{2^{-\frac{\nu }{2}}e^{-\frac{z^{2}}{4}}}{\Gamma (\frac{1}{2}+\nu )}\sum_{k=0}^{\infty }\frac{\Gamma \left(-\nu +k\right)z^{2k}}{\Gamma (-\nu )(2k-1)!!k!}, \end{array}$$

where we use ${\left(x\right)}_{n}=\frac{\Gamma (x+n)}{\Gamma (x)}$ and ${\left(\frac{1}{2}\right)}_{n}=\frac{(2n-1)!!}{2^{n}}$. Then, we can write that

(A17) $$\begin{array}{ccc} D_{-(2n-2m+2)}\left(\frac{iXx_{\omega }\sqrt{\nu +i\mu x_{\omega }^{2}}}{\sqrt{\nu }\sqrt{\nu^{2}+\mu^{2}x_{\omega }^{4}}}\right) & = & \frac{2^{n-m+1}e^{\frac{X^{2}x_{\omega }^{2}\left(\nu +i\mu x_{\omega }^{2}\right)}{4\nu (\nu^{2}+\mu^{2}x_{\omega }^{4})})}}{\Gamma (\frac{1}{2}-\left(2n-2m+2\right))}\\ & \times & \sum_{k=0}^{\infty }\frac{\Gamma \left(2n-2m+2+k\right){\left(\frac{-X^{2}x_{\omega }^{2}\left(\nu +i\mu x_{\omega }^{2}\right)}{\nu (\nu^{2}+\mu^{2}x_{\omega }^{4})}\right)}^{k}}{\Gamma (2n-2m+2)(2k-1)!!k!}, \end{array}$$

holds. It is easy to see that the complex conjugate of the latter function is

(A18) $$\begin{array}{ccc} D_{-(2n-2m+2)}^{★}\left(\frac{iXx_{\omega }\sqrt{\nu +i\mu x_{\omega }^{2}}}{\sqrt{\nu }\sqrt{\nu^{2}+\mu^{2}x_{\omega }^{4}}}\right) & = & D_{-(2n-2m+2)}\left(\frac{iXx_{\omega }\sqrt{\nu -i\mu x_{\omega }^{2}}}{\sqrt{\nu }\sqrt{\nu^{2}+\mu^{2}x_{\omega }^{4}}}\right). \end{array}$$

Then, the tomogram is the following

(A19) $$\begin{array}{ccc} & & W_{n}\left(X|0,\mu ,\nu \right)\;\;=\;\;\frac{1}{\pi }\frac{{\left(\left(2n+1\right)!\right)}^{2}}{n!(n+\frac{1}{2})!}\frac{\nu^{2n+1}x_{\omega }}{{\left(\nu^{2}+\mu^{2}x_{\omega }^{4}\right)}^{n+1}}e^{-\frac{X^{2}x_{\omega }^{2}}{2\left(\nu^{2}+\mu^{2}x_{\omega }^{4}\right)}}\\ & \times & [\sum_{m_{1},m_{2}=0}^{n}\frac{{\left(-1\right)}^{m_{1}+m_{2}}}{m_{1}!m_{2}!}{\left(\frac{x_{\omega }^{2}}{16\nu }\right)}^{m_{1}+m_{2}}{\left(\nu -i\mu x_{\omega }^{2}\right)}^{m_{1}}{\left(\nu +i\mu x_{\omega }^{2}\right)}^{m_{2}}\\ & \times & D_{-(2n-2m_{1}+2)}\left(\frac{iXx_{\omega }\sqrt{\nu +i\mu x_{\omega }^{2}}}{\sqrt{\nu }\sqrt{\nu^{2}+\mu^{2}x_{\omega }^{4}}}\right)D_{-(2n-2m_{2}+2)}\left(\frac{iXx_{\omega }\sqrt{\nu -i\mu x_{\omega }^{2}}}{\sqrt{\nu }\sqrt{\nu^{2}+\mu^{2}x_{\omega }^{4}}}\right)]. \end{array}$$

## Appendix C. Tomogram for the Pseudoharmonic Oscillator for Any *A*

Let us find the tomogram for (53). According to (6), it can be written as

(A20) $$\begin{array}{ccc} W(X|\mu ,\nu ) & = & \frac{1}{2\pi |\nu |}\frac{1}{x_{\omega }}\left[\frac{2n!}{\Gamma (n+\eta +1)}\right]\\ & \times & |\int_{0}^{\infty }{\left(\frac{y}{x_{\omega }}\right)}^{\eta +\frac{1}{2}}\exp\left(-\frac{1}{2}\frac{y^{2}}{x_{\omega }^{2}}\right)L_{n}^{\left(\eta \right)}\left(\frac{y^{2}}{x_{\omega }^{2}}\right)\exp\left(\frac{i\mu }{2\nu }y^{2}-\frac{iX}{\nu }y\right)dy{|}^{2}. \end{array}$$

Changing the variables to $p=\frac{1}{2}-\frac{i\mu }{2\nu }x_{\omega }^{2}$, $q=\frac{iX}{\nu }x_{\omega }$, we have

(A21) $$\begin{array}{c} W_{n}\left(X|\mu ,\nu \right)=\frac{x_{\omega }}{2\pi |\nu |}\left[\frac{2n!}{\Gamma (n+\eta +1)}\right]|\int_{0}^{\infty }t^{\eta +\frac{1}{2}}\exp\left(-pt^{2}-qt\right)L_{n}^{\left(\eta \right)}\left(t^{2}\right)dt{|}^{2}. \end{array}$$

Similarly to the previous subsection, we can use the confluent hypergeometric function representation:

(A22) Ln(η)(t2)=(η+1)nnF11(−n;η+1;t2)=(η+1)nn∑k=0∞(n)kt2k(η+1)kk!.

Then, the tomogram can be written as

$$\begin{array}{c} W_{n}\left(X|\mu ,\nu \right)=\frac{x_{\omega }}{2\pi |\nu |}\left[\frac{2n!}{\Gamma (n+\eta +1)}\right]|\frac{{\left(\eta +1\right)}_{n}}{n}\sum_{k=0}^{\infty }\frac{{\left(n\right)}_{k}}{{\left(\eta +1\right)}_{k}k!}\int_{0}^{\infty }t^{2k+\eta +\frac{1}{2}}\exp\left(-pt^{2}-qt\right)dt{|}^{2}. \end{array}$$

We use (A11) to write the closed form of the tomogram:

(A23) $$\begin{array}{ccc} & & W_{n}\left(X|\mu ,\nu \right)=\frac{x_{\omega }}{2\pi |\nu |}\left[\frac{2n!}{\Gamma (n+\eta +1)}\right]\exp\left(-\frac{X^{2}x_{\omega }^{2}}{2(\nu^{2}+\mu^{2}x_{\omega }^{4})}\right){\left(\frac{{\left(\eta +1\right)}_{n}}{n}\right)}^{2}\\ & \times & |\sum_{k=0}^{\infty }\frac{{\left(n\right)}_{k}}{{\left(\eta +1\right)}_{k}k!}\Gamma \left(2k+\eta +\frac{3}{2}\right){\left(\frac{\nu -i\mu x_{\omega }^{2}}{\nu }\right)}^{-(k+\frac{\eta }{2}+\frac{3}{4})}D_{-(2k+\eta +\frac{3}{2})}\left(\frac{iXx_{\omega }\sqrt{\nu +i\mu x_{\omega }^{2}}}{\sqrt{\nu }\sqrt{\nu^{2}+\mu^{2}x_{\omega }^{4}}}\right){|}^{2}. \end{array}$$
